# Extracellular vesicle-mediated communication between CD8^+^ cytotoxic T cells and tumor cells

**DOI:** 10.3389/fimmu.2024.1376962

**Published:** 2024-03-18

**Authors:** Zeyu Huang, Xuehui Liu, Qinghao Guo, Yihang Zhou, Linlin Shi, Qingjin Cai, Shupei Tang, Qin Ouyang, Ji Zheng

**Affiliations:** ^1^ Department of Urology, Urologic Surgery Center, Xinqiao Hospital, Third Military Medical University (Army Medical University), Chongqing, China; ^2^ Department of Medicinal Chemistry, College of Pharmacy, Third Military Medical University, Chongqing, China; ^3^ Department of Shigatse Branch, Xinqiao Hospital, Third Military Medical University, Shigatse, China

**Keywords:** CD8^+^ T cell, extracellular vesicles, tumor, tumor microenvironment, immunity

## Abstract

Tumors pose a significant global public health challenge, resulting in numerous fatalities annually. CD8^+^ T cells play a crucial role in combating tumors; however, their effectiveness is compromised by the tumor itself and the tumor microenvironment (TME), resulting in reduced efficacy of immunotherapy. In this dynamic interplay, extracellular vesicles (EVs) have emerged as pivotal mediators, facilitating direct and indirect communication between tumors and CD8^+^ T cells. In this article, we provide an overview of how tumor-derived EVs directly regulate CD8^+^ T cell function by carrying bioactive molecules they carry internally and on their surface. Simultaneously, these EVs modulate the TME, indirectly influencing the efficiency of CD8^+^ T cell responses. Furthermore, EVs derived from CD8^+^ T cells exhibit a dual role: they promote tumor immune evasion while also enhancing antitumor activity. Finally, we briefly discuss current prevailing approaches that utilize functionalized EVs based on tumor-targeted therapy and tumor immunotherapy. These approaches aim to present novel perspectives for EV-based tumor treatment strategies, demonstrating potential for advancements in the field.

## 1 Introduction

Cancer represents a significant global public health concern, with rapidly increasing incidence and mortality rates worldwide (1, 2). Currently, immunotherapy has emerged as a cutting-edge field in oncology, providing highly targeted and potent treatment options (3, 4). Immunotherapy harnesses the patient’ s immune system to combat tumors, presenting several advantages over conventional therapies, including rapid initiation, fewer side effects, and a broader range of treatment possibilities (5). CD8^+^ T lymphocytes play a critical role in immunotherapy (6, 7), serving as powerful effectors in antitumor immune responses and exhibiting the capacity to recognize and eliminate malignant cells (7–9). Once stimulated, antigen-specific CD8^+^ T cells undergo clonal expansion and differentiate into cytotoxic CD8^+^ T cells (CTLs), which then migrate throughout the body to identify and eradicate cancer cells (10, 11).

Although significant progress has been achieved in the field of immunotherapy, a substantial proportion of patients remain unresponsive to these treatment. Recent evidence from both human and murine cancer studies suggests that tumors and the tumor microenvironment (TME) play a crucial role in reshaping CD8^+^ T cells through various suppressive signals (12, 13). The TME refers to the ecosystem surrounding a tumor inside the body, comprising dendritic cells (DCs), tumor-associated macrophages (TAMs), myeloid-derived suppressor cells (MDSCs), regulatory T cells (Tregs), and other components that contribute to the immunosuppression of CD8^+^ T cells (14–17). However, the specific regulatory mechanisms through which tumors and the TME influence dysfunctional CD8^+^ T cells are still not fully understood.

Extracellular vesicles (EVs), which are released by most cells, are membranous vesicle-like structures comprising a lipid bilayer, and containing proteins, lipids, and nucleic acids (including DNA, mRNA, and noncoding RNA) (18–20). Based on their biogenesis and size, EVs are classified into three different categories (**Table 1**). Exosomes, with diameters ranging from 30 to 150 nm, originate and are released from endosomal compartments. Microvesicles (ectosomes or microparticles), sized between 100 and 1000 nm, bud directly from the plasma membrane. Apoptotic bodies, characterized by their larger sizes spanning from 50-5000 nm, are released from apoptotic cells (24, 43, 44). EVs play a crucial role in intercellular communication, facilitating the transport of various bioactive molecules and are considered as super vital in tumor progression and antitumor immune responses (45, 46). A growing body of research has been dedicated to investigating the involvement of EVs in tumors, primarily focusing on elucidating the direct impact of tumor-derived EVs (TDEs) on CD8^+^ T cells (47). Nevertheless, these studies often overlook the vital indirect regulatory role of the TME in this intricate process. The current review aims to address this gap by presenting recent study findings on EV-mediated direct and indirect bidirectional communication between CD8^+^ T cells and tumors. Specifically, we provide an overview of the latest research regarding the direct regulation (bioactive molecules inside or on the surface of EVs) of CD8^+^ T cells by TDEs. We also review the indirect regulatory effects of TDEs on immune cells within the TME (including DCs, TAMs, and MDSCs). Additionally, we summarize the bidirectional regulatory effects of CD8^+^ T cell-derived EVs on tumor cells. Furthermore, we explore the use of engineered EVs to activate CD8^+^ T cells (tumor-targeted therapy and tumor immunotherapy) and enhance their ability to kill tumors. The review offers a comprehensive insight into the interactions among EVs, CD8^+^ T cells, the TME, and tumors. Additionally, it provides a unique perspective on the therapeutic potentials of EVs in the treatment of tumors.

**Table 1:** Categories of extracellular vesicles based on size and biogenesis.

Type	Exosome	Microvesicles	Apoptotic bodies
Diameter	30-150 nm	100-1000 nm	50-5000 nm
Biogenesis	Released by polyvesicles (late endosomes with internal vesicles) fused to the plasma membrane (21)	Germinated on the plasma membrane (22, 23)	Released by apoptotic cells (24, 25)
Function	Oncogenic agents delivery (26), transmit traits (like drug resistance) (27, 28), promote tumor invasion and metastasis (26, 29), induce the formation of microvasculature or thrombus; influence stromal cells and lymphocytes in TME (30–34).	Facilitate communication (35, 36); induce invasion (37–39); promote angiogenesis on endothelial cells (40, 41).	Phosphatidylserine ectropion, which may contain fragmented DNA, horizontal metastasis of oncogenes (42).

TME, tumor microenvironment.

## 2 Effects of TDEs on CD8^+^ T cells

### 2.1 Direct effects

**Figure 1:**
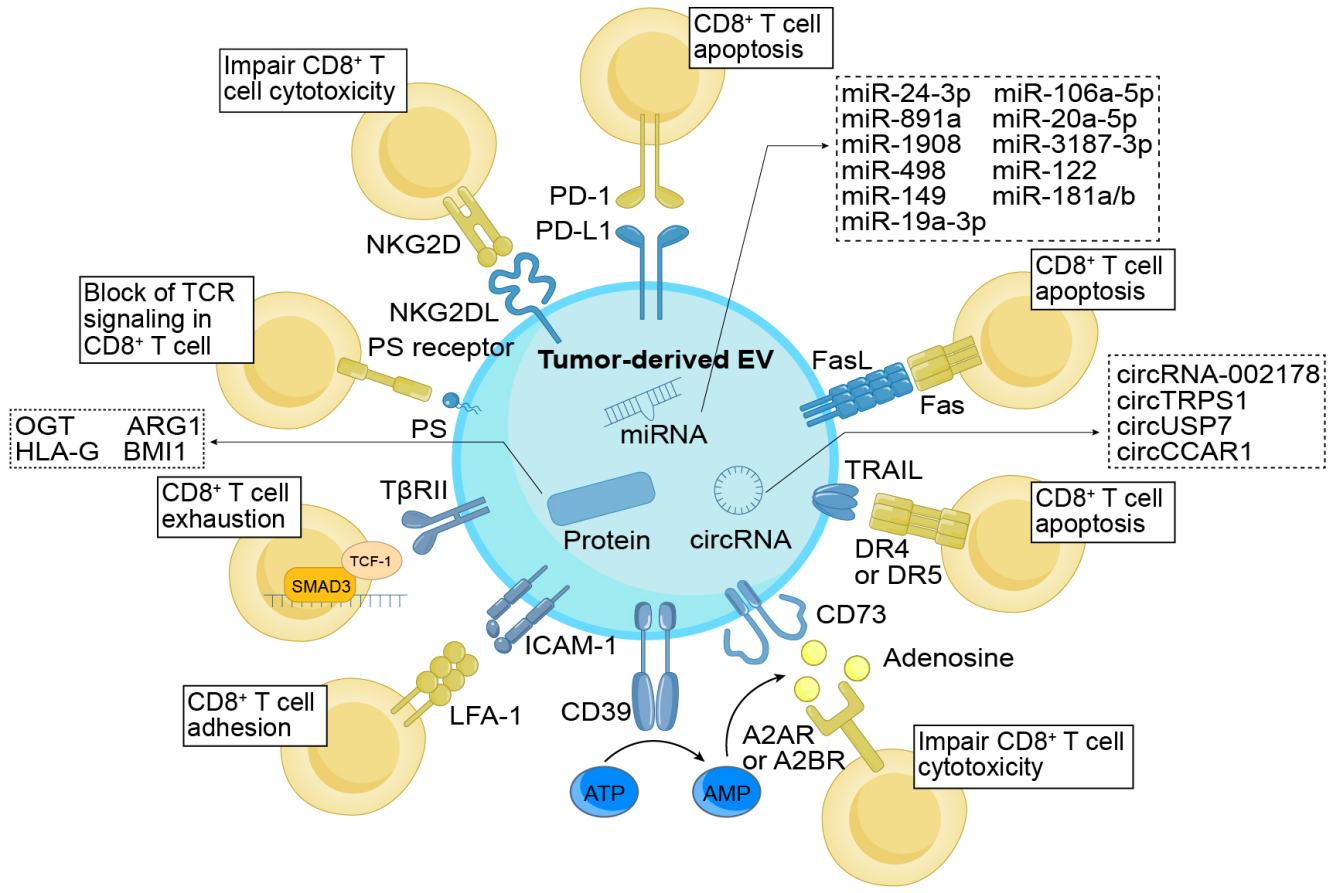
Direct immunomodulatory function of TDEs on CD8^+^ T cells. TDEs exert regulatory control over CD8^+^ T cells through their cargo and surface molecules. These surface molecules, including PD-L1, FasL, TRAIL, PS, and NKG2DL, directly interact with cognate ligands or receptors, impeding the activation and cytotoxicity of CD8^+^ T cells while promoting apoptosis. In addition, TDEs indirectly modulate CD8^+^ T cell function by influencing molecules like CD39 and CD73, which convert ATP into adenosine, thereby suppressing the cytotoxic abilities of CD8^+^ T cells. ICAM-1, through its interaction with LFA-1, promotes the adhesion of TDEs and CD8^+^ T cells. Additionally, TβRII induces CD8^+^ T cell exhaustion by activating the TGF-β signaling pathway. Simultaneously, the cargo enclosed within TDEs also holds significant importance in immune regulation. The TDEs contained miRNAs, circRNAs, and proteins function by suppressing the proliferation (e.g., miR-24-3p, ARG1, etc.), differentiation (e.g., miR-891a, miR-106a-5p, etc.), activation (e.g., miR-3187-3p, etc.), and cytotoxicity (e.g., HLA-G, etc.) of CD8^+^ T cells, while facilitating cellular exhaustion (e.g., circUSP7, OGT, etc.). As a result, they effectively undermine the anti-tumor effects of CD8^+^ T cells. TDEs, tumor-derived extracellular vesicles; PD-L1, programmed cell death ligand-1; FasL, Fas ligand; TRAIL, tumor necrosis factor-related apoptosis-inducing ligand; ICAM-1, intercellular adhesion molecule-1; TβRII, TGF-β type II receptor; PS, phosphatidylserine; NKG2DL, NKG2D ligand; PD-1, programmed cell death protein 1; LFA-1, lymphocyte function-associated antigen-1; TCR, T cell receptor; OGT, O-GlcNAc transferase; ARG1, arginase-1; HLA-G, human leukocyte antigen-G.

Tumors have the ability to evade the immune system’s antitumor response by specifically targeting CD8^+^ T cells that infiltrate the tumor. They employ various mechanisms to accomplish this evasion, such as inhibiting, rendering inactive, or deceiving CTLs, ultimately resulting in the evasion of cancer cells and, in some instances, triggering apoptosis in the T cells themselves (48). With advancements in TDE research, increasing attention is being directed toward comprehending the role of TDEs as communication messengers in inhibiting CD8^+^ T cells. In this context, we herein explore the mechanisms through which tumor cells directly impact CD8^+^ T cells via TDEs, with a focus on both the contents and membrane molecules involved (**Figure 1**).

#### 2.1.1 Components

Tumor cells employ EVs as carriers to transport a diverse range of cargo, including nucleic acids and proteins, to CD8^+^ T cells. Interestingly, TDEs containing micro RNAs (miRNAs) can actively participate in modulating of CD8^+^ T cell immune responses. Ye et al. identified five upregulated miRNAs—miR-24-3p, miR-891a, miR-106a-5p, miR-20a-5p, and miR-1908—from exosomes derived from nasopharyngeal carcinoma cells. They demonstrated that these miRNAs could downregulate the MARK1 signaling pathway, thereby affecting the proliferation and differentiation of CD8^+^ T cells (49). In EVs derived from melanoma, specific miRNAs, including miR-3187-3p, miR-498, miR-122, miR-149, and miR-181a/b, were found to downregulate T cell receptor (TCR) signaling and the secretion of tumor necrosis factor-α (TNF-α) in CD8^+^ T cells (50). Furthermore, miR-19a-3p was identified within EVs derived from leukemic cells and subsequently internalized by CD8^+^ T cells. This internalization induced immunosuppression through the SLC6A8-mediated creatine import (51). These studies provide evidence highlighting the significant roles that miRNAs play in the immunoregulation of CD8^+^ T cells.

Circular RNAs (circRNAs), an endogenous class of noncoding RNAs, play diverse roles and are integral components in various biological processes, particularly in tumor development and progression (52). Recently, circRNAs within TDEs have emerged as crucial signaling molecules involved in intercellular communication, notably influencing CD8^+^ T cell function modulation. In lung adenocarcinoma, it was observed that TDEs transported circRNA-002178 to CD8^+^ T cells, resulting in an upregulation of programmed cell death protein-1 (PD-1) expression and subsequently inducing CD8^+^ T cell exhaustion (53). Additionally, the presence of circTRPS1 in exosomes released by bladder cancer cells was implicated in inducing CD8^+^ T cell exhaustion by regulating intracellular reactive oxygen species (ROS) balance along the circTRPS1/miR141-3p/GLS1 pathway (54). In non-small cell lung cancer, TDEs containing circUSP7 were found to play crucial roles in promoting CD8^+^ T cell exhaustion through the miR-934/SHP2 axis, ultimately leading to immunosuppression (55). Following the absorption of exosomes derived from hepatocellular carcinoma cells containing circCCAR1, CD8^+^ T cells exhibited elevated exhaustion levels. This was primarily attributed to the inhibition of PD-1 ubiquitin degradation by these exosomes, consequently impairing CD8^+^ T cell function (56).

In addition to RNAs, TDEs are recognized for transferring proteins to tumor cells, thereby influencing CD8^+^ T cells. In cholangiocarcinoma, TDEs containing BMI1 impede chemokine recruitment by CD8^+^ T cells, accomplishing this inhibition through the enhancement of inhibitory H2A ubiquitination in tumor cells via autocrine or paracrine mechanisms (57). Proteins enclosed within TDEs have the capacity to modulate CD8^+^ T cell behaviors. O-GlcNAc transferase, for instance, enhances tumor growth and migration (58). According to Yuan et al., exosomal O-GlcNAc transferase from esophageal carcinoma stem cells is absorbed by CD8^+^ T cells, resulting in an upregulation of PD-1 in these cells and thereby protecting esophageal carcinoma stem cells (59). Furthermore, research has demonstrated that EVs containing arginase-1 directly impede CD8^+^ T cell proliferation by reducing L-arginine levels (60). Moreover, proteins can induce changes in CD8^+^ T cell phenotypes. Human leukocyte antigen-G (HLA-G) is a recently discovered IC inhibitor that binds to immunoglobulin-like transcript (ILT)-2 and ILT-4, which are inhibitory receptors found on immune cells that play an essential role in facilitating tumor growth (61). Recent evidence indicates that HLA-G EVs enhance the immunosuppressive characteristics of ILT-2-negative CD8^+^ T cells in breast cancer (BC). However, the specific impact of HLA-G on TDEs is yet to be explored (62).

Overall, gaining a comprehensive understanding of the precise mechanisms through which TDEs transport cargo to CD8^+^ T cells holds the potential to reveal novel avenues for cancer treatment.

#### 2.1.2 Membrane molecules

The membrane molecules of TDEs play a pivotal role in the tumor immune response, encompassing functions such as immune evasion, modulation of immune cell polarization and function, and suppression of the initiation of antitumor immune responses. Recently, there has been an increasing interest in understanding the regulation of TDE membrane molecules in CD8^+^ T cells, with a particular focus on ICs as a the most noteworthy aspect. One of the most significant mechanisms in ICs is immunosuppression mediated by the PD-1/PD-L1 pathway. PD-L1 expression is widely observed on immune cells, particularly CD8^+^ T cells, while residing predominantly on the surface of various tumor cells (63). The interaction between PD-L1 and PD-1 can lead to the inhibition of immune function in CD8^+^ T cells. Recent research has demonstrated the presence of PD-L1 on the surface of TDEs from various malignancies, including head and neck squamous cell carcinoma, lung cancer, hypopharyngeal cancer, melanoma, and nasopharyngeal carcinoma (64–69). Notably, the expression of PD-L1 on the surface of TDEs is also regulated by various cytokines, such as interferon-γ (IFN-γ), transforming growth factor-β (TGF-β), and type I interferon (IFN-I) (70–74). Apart from PD-L1, other ICs have also been identified on the surface of TDEs. A previous study revealed that TDEs from prostate cancer carry Fas ligand (FasL), inducing the apoptosis of CD8^+^ T cells (75). Clayton et al. demonstrated that exosomes carry CD39 and CD73, resulting in the inhibition of CD8^+^ T cells by converting ATP into adenosine (76). Additionally, Azambuja et al. found that TDEs from glioblastoma express FasL, TRAIL, CD39, and CD73, along with a few immunostimulatory proteins, leading to the inhibition of TNF-α and INF-γ released from CD8^+^ T cells and the induction of apoptosis (77). Overall, these findings indicate that investigating the ICs present in EVs can potentially provide crucial insights into the complex mechanisms through which tumor cells evade immune responses mediated by CD8^+^ T cells.

In addition to ICs, other molecules present on the surface of TDEs actively participate in suppressing the immune function of CD8^+^ T cells. A crucial regulatory factor in inducing immune escape is the signal transduction of TGF-β. According to Xie et al., TDEs express TGF-β type II receptor (TβRII) on their surface in BC, and by delivering TβRII to CD8^+^ T cells, they stimulate TGF-β signal transduction, leading to the exhaustion of these T cells (78). The activating cytotoxicity receptor NKG2D is predominantly expressed on immune cells, including CD8^+^ T cells, whereas the soluble form of NKG2D ligand (NKG2DL) is utilized by tumors to evade immune responses (79, 80). Clayton et al. found that NKG2DL was presented on the surface of human TDEs and could downregulate the expression of NKG2D receptors on CD8^+^ T cells (81). Similarly, Lundholm et al. discovered that NKG2DL was also presented on the surface of TDEs derived from prostate cancer (82). Furthermore, adhesion molecules can facilitate interactions between TDEs and CD8^+^ T cells. Zhang et al. revealed that ICAM-1 was involved in mediating the adhesion between TDEs and CD8^+^ T cells through its interaction with LFA-1 on the surface of the CD8^+^ T cells, thus being indispensable for the functionality of exosomal PD-L1 in immunomodulation (83).

Apart from immunosuppressive proteins, the lipids present in EVs also exert inhibitory effects on the functionality of CD8^+^ T cells. The identification of phosphatidylserine on the surface of TDEs in ovarian tumors has been associated with the induction of a blockade in TCR signaling in CD8^+^ T cells. This discovery underscores the potential importance of targeting TDE-associated phosphatidylserine as a therapeutic approach for tumor treatment (84).

Based on the above findings from previous studies, TDEs not only carry transport cargoes within their lumen but can also express them on their surface, facilitating the efficient delivery of immunosuppressive cargoes to CD8^+^ T cells. Consequently, a more in-depth exploration of the specific molecules carried by TDEs is essential for a thorough understanding of the mechanisms underlying CD8^+^ T cell exhaustion.

### 2.2 Indirect effects through cells in the TME

**Figure 2:**
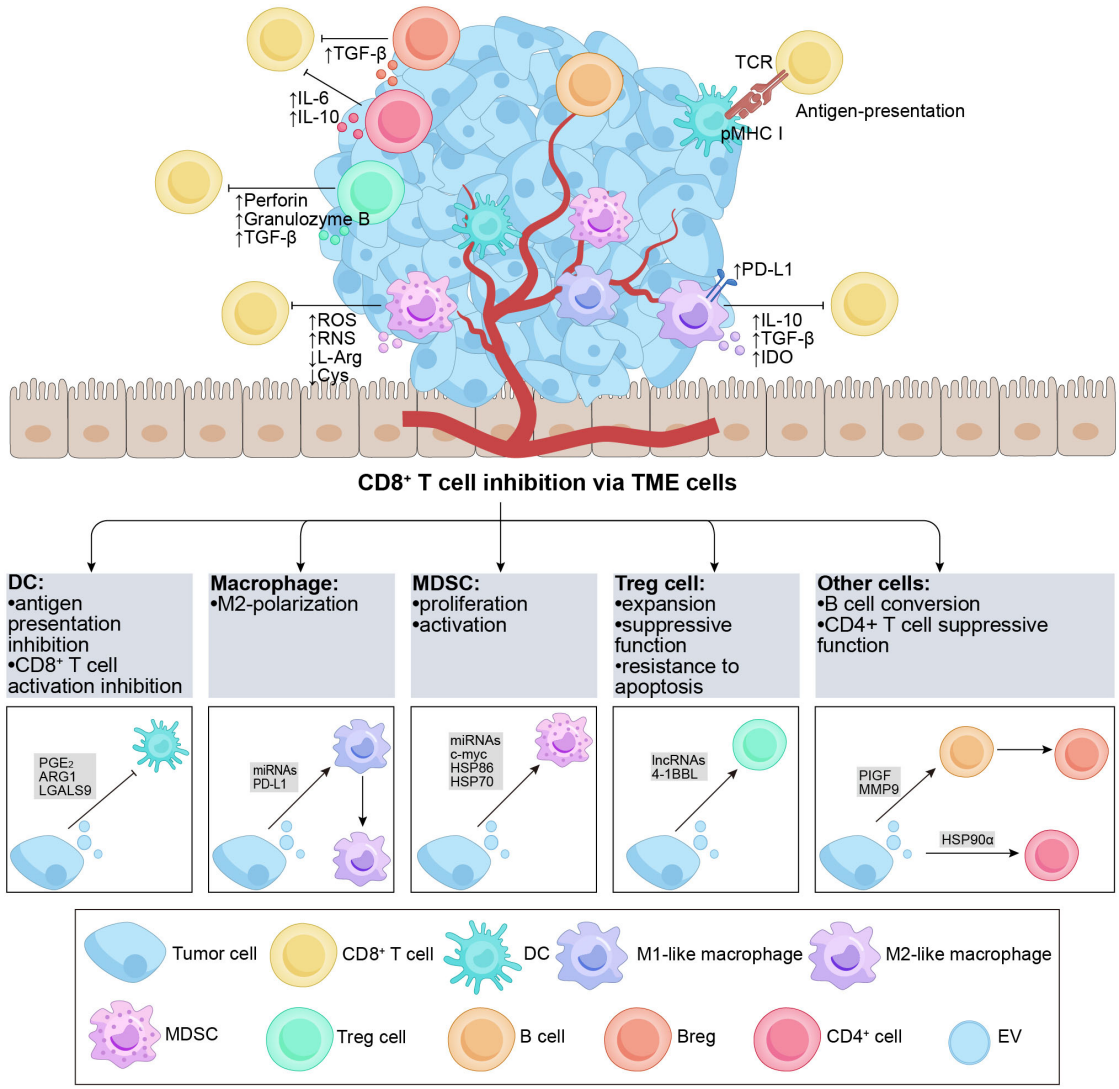
Overview of TDEs indirectly regulate CD8^+^ T cells via the TME cells. The TME contains various subgroups of immune cells, such as DCs, B cells, macrophages, Tregs, MDSCs, etc. The figure demonstrates the regulatory effects of different cell subgroups on CD8^+^ T cells. It is noteworthy that TDEs transport a diverse range of regulatory molecules that interact with immune cells in the TME through various pathways, resulting in inhibitory effects on CD8^+^ T cells. DCs possess the capability to effectively capture and present antigens. TDEs impede the activation of DCs and the presentation of antigens (e.g., PGE_2_, ARG1, etc.), thereby suppressing the activation of CD8^+^ T cells. Furthermore, TDEs can also hinder the functionality of CD8^+^ T cells by influencing cell polarization (e.g., PIGF, MMP9, miR-1231-5p, PD-L1, etc.) in M2-like macrophages and Bregs, ultimately promoting tumor growth. Additionally, TDEs proficiently inhibit anti-tumor immune responses by augmenting the activation, proliferation, and functionality of Tregs and MDSCs (e.g., 4-1BBL, HSP-86, HSP-70, miR-21, etc.). TDEs, tumor-derived extracellular vesicles; TME, tumor microenvironment; DC, dendritic cell; MDSC, myeloid-derived suppressor cell; Treg, regulatory T cell; PGE_2_, prostaglandin E^+^_2_; TCR, T cell receptor; pMHC I, peptide major histocompatibility complex class I; PD-L1, programmed cell death ligand-1; IL, interleukin; TGF-β, transforming growth factor-β; IDO, indoleamine 2,3-dioxygenase; ROS, reactive oxygen species; RNS, reactive nitrogen species; L-Arg, L-Arginine; ARG1, arginase-1; Cys, Cystine; PlGF, placenta growth factor; MMP9, matrix metalloprotease-9; HSP, heat shock protein.

The TME is a dynamic and intricate ecosystem during the process of tumorigenesis, comprising various immune cells (such as T and B lymphocytes, TAMs, DCs, natural killer cells, neutrophils, and MDSCs), stromal cells, the extracellular matrix, secreted molecules, as well as the blood and lymphatic vascular networks (85). The TME actively participates in the cancer-immune cycle and significantly influences tumor growth, advancement, and treatment outcomes (86). In the context of tumor therapy, the TME exerts immunomodulatory effects by targeting CD8^+^ T cells (87). TDEs also play a substantial role in immunomodulation within the TME (88), indirectly impacting CD8^+^ T cells through the components of the TME. In the subsequent discussion, we focus on the key immune cell populations within the TME and their role in mediating the indirect effects of TDEs on CD8^+^ T cells (**Figure 2**).

#### 2.2.1 DCs

DCs play a crucial role in initiating and controlling both innate and adaptive immune responses, serving as a distinct group of antigen-presenting cells (89). DCs possess the capability to efficiently capture and cross-present tumor antigens, activating CD8^+^ T cells and impeding tumor growth and progression (90, 91). Within the TME, TDEs indirectly suppress the immune response of CD8^+^ T cells by compromising the antigen-presenting function of DCs (88, 92). A study revealed that EVs derived from prostate cancer cells containing prostaglandin E^+^_2_ could enhance the expression of the CD73 molecule on the surface of DCs, inhibiting the cross-presentation of tumor-associated antigens by DCs and impairing the activation of CD8^+^ T cells (93). Similarly, EVs from ovarian cancer cells, containing arginase-1, could be internalized by DCs, impairing the activation of CD8^+^ T cells (60). Another study highlighted that exosomal LGALS9 from glioblastoma multiforme tumors could bind to the TIM3 receptor of DCs, inhibiting DC antigen presentation and suppressing CD8^+^ T cell activation (94). Furthermore, Lewis lung cancer cells (with EGFR E746-A750 deletion) secreted exosomes that transferred active E746-A750 deletion to the surface of DCs, resulting in suppressed DC function and enhanced tumor growth (95). Overall, tumor antigens carried by TDEs hinder the cross-presentation of DCs and the expression of maturation markers, ultimately impairing CD8^+^ T cell activation. This suggests that TDEs participate in immunomodulation within DCs as tumors progress, and the evaluation of the DC phenotype holds prognostic value for patients.

#### 2.2.2 Macrophages

Macrophages are versatile cells that play essential roles in modulating immune responses and tumor development (96, 97). In tumor tissues, infiltrating macrophages, known as TAMs, can undergo polarization, resulting in the formation of M1-like macrophages that exhibit antitumor effects and M2-like macrophages that facilitate tumor growth by inhibiting CD8^+^ T cells (98, 99). M2 TAMs express PD-L1 and release inhibitory cytokines such as IL-10 and TGF-β, causing exhaustion and directly suppressing CD8^+^ T cell function. Moreover, M2 TAMs hinder CD8^+^ T cell proliferation and activity through the indoleamine-2,3-dioxygenase pathway (100). The reported findings suggest that TDEs have the potential to compromise the cytotoxic response of CD8^+^ T cells by inducing M2 polarization in macrophages. Specifically, exosomal miR-1231-5p and miR-92b-3p derived from bladder cancer cells activate the PTEN/AKT/STAT3/6 pathway, leading to the polarization of macrophages into the M2 phenotype. Consequently, this polarization inhibits the proliferation of CD8^+^ T cells (101). Meanwhile, one study reported that after the internalization of colorectal cancer-derived exosomes by macrophages, miR-21-5p and miR-200a could trigger the polarization of macrophages into an M2-like phenotype and upregulate the expression of PD-L1. This effect was achieved through the regulation of the PTEN/AKT and SCOS1/STAT1 pathways. Consequently, reduced activity of CD8^+^ T cells was observed, leading to enhancing tumor growth (102).

Notably, TDEs not only regulate the M2 differentiation of TAMs but also inhibit the function of CD8^+^ T cells by promoting the expression of PD-1/PD-L1 on the membrane of M2 TAMs. Exosomes derived from gastric cancer cells induce the differentiation of monocytes into PD-1^+^ TAMs. Upon interaction with PD-L1^+^ cells, PD-1^+^ TAMs produce IL-10 and impair the functions of CD8^+^ T cells (103). Another study reported that upon uptake of tumor cell-released autophagosomes (TRAPs), monocytes activate the MyD88-p38-STAT3 pathway via TLR4 to express an M2-like phenotype, leading to PD-L1 upregulation and IL-10 expression. This further inhibits the function of CD8^+^ T cells and enhances tumor progression (104).

Multiple factors contribute to the regulation of PD-1/PD-L1 expression in TAMs via TDEs, including endoplasmic reticulum (ER) stress, ROS, and proteins. When hepatocellular carcinoma cells are exposed to ER stress, it leads to the secretion of exosomal miR-23a-3p. This specific miRNA is transferred to M2 TAMs and enhances the expression of PD-L1 through the PTEN/AKT pathway, thereby exerting an inhibitory effect on CD8^+^ T cell-mediated immune responses (105). Additionally, ER stress-induced BC-derived exosomes containing miR-27a-3p can upregulate PD-L1 in M2 TAMs via the PTEN/AKT/PI3K pathway, ultimately promoting the inhibition of CD8^+^ T cells (106). In ovarian cancer, a high level of ROS can reduce the uptake of tumor-derived exosomal miR-155-5p by macrophages, resulting in the upregulation of PD-L1 and subsequent suppression of CD8^+^ T cells (107). Moreover, Golgi membrane protein 1 can inhibit the expression of Rab27b, promoting COP9 signalosome 5-mediated deubiquitination of PD-L1 and enhancing the transport of exosomal PD-L1 into M2 TAMs, ultimately causing increased suppression of CD8^+^ T cells (108).

Overall, TDEs have the ability to promote M2 polarization of macrophages and utilize immunosuppressive molecules to hinder CD8^+^ T cell activation.

#### 2.2.3 MDSCs

MDSCs, (pathologically activated neutrophils) and monocytes, play a role in suppressing CD8^+^ T cell-mediated immune responses through various mechanisms, including the production of ROS and reactive nitrogen species as well as the depletion of L-arginine and cystine in the TME (109). MDSCs are essential components in tumor development, metastasis, and resistance to treatment. Within the TME, TDEs substantially contribute to promoting the proliferation and activation of MDSCs, thereby contributing to the suppression of CD8^+^ T cells (88). For example, in acute myeloid leukemia, MUC1 within TDEs promotes the expression of c-myc by inhibiting the expression of miR-34a, inducing the proliferation of MDSCs (110). Additionally, research has shown that hypoxia stimulates the release of glioma-derived exosomes, leading to the expression of miR-10a and miR-21 within these exosomes. Consequently, RAR-related orphan receptor alpha and PTEN are activated, resulting in the expansion and activation of MDSCs (111). Moreover, melanoma-derived EVs activate TLR4 on myeloid cells by inducing heat shock protein (HSP) 86 within the exosomes. This activation triggers the NF-κB pathway and upregulates PD-L1 expression, ultimately promoting the conversion of immature myeloid cells into MDSCs (112). ​In a separate laboratory study, exosomes derived from renal cancer were found to stimulate the proliferation and activation of MDSCs. This effect was attributed to the binding of HSP70 within these EVs to TLR2 receptors expressed on MDSCs. Consequently, this interaction hindered the cytotoxic impact of CD8^+^ T cells and facilitated the advancement of tumor growth (113). Moreover, a study revealed that miR-9 and miR-181a derived from exosomes in BC had the ability to activate the JAK/STAT signaling pathway. This activation occurred through the targeting of SOCS3 by miR-9 and PIAS3 by miR-181a. Consequently, these molecular interactions promoted early-stage MDSC expansion, inhibited CD8^+^ T cell proliferation, and induced CD8^+^ T cell apoptosis, ultimately facilitating tumor growth (114). Another study demonstrated that exosomes derived from BC cells could induce the differentiation of bone marrow cells into MDSCs. Notably, these exosomes significantly inhibited the proliferation of CD8^+^ T cells through the downregulation of CXCR4 expression and activation of the STAT3 signaling pathway (115). In summary, TDEs play a pivotal role in regulating the differentiation and phenotype of MDSCs by transporting specific cargo to these cells. This process leads to the amplification and activation of MDSCs, thereby providing further support for tumor progression.

#### 2.2.4 Tregs

Tregs play a critical role in maintaining immune balance within the TME. They help prevent excessive immune reactions against normal body tissues (self-antigens) and hinder the immune system’s capacity to initiate a potent antitumor response (116, 117). Tregs primarily limit the activity of CD8^+^ T cells, employing various inhibitory mechanisms, including the direct killing of CD8^+^ T cells by activated Tregs through the release of substances like perforin or granzyme B. Additionally, Tregs can release TGF-β, which directly hinders the activation of CD8^+^ T cells (118).

Several studies have suggested that TDEs indirectly impede the functioning of CD8^+^ T lymphocytes by modulating Tregs. Within the TME, TDEs utilize receptor-ligand signaling interactions on their surface to facilitate the expansion of Tregs, enhance their suppressor function, and increase their resistance to apoptosis (119–121). In BC, γδ T cells were identified as the predominant lymphocyte population infiltrating the tumor, playing a critical role in immunosuppression (122). The study revealed that CD73^+^ γδT1 cells were the major subset of T lymphocytes with regulatory characteristics in BC. In another study, TDEs influenced the activation of the TGF-β1/SMAD5 pathway through the SNHG16/miR-16-5p/SMAD5 regulatory axis. This led to the upregulation of CD73 expression, which, in turn, inhibited immune function via adenosine signaling in γδ1 T cells. Consequently, CD73^+^γδT1 cells exhibited the ability to suppress the secretion of perforin and granzyme B by CD8^+^ T cells (123). TDEs possess the ability to induce the differentiation of Tregs and facilitate the acquisition of a suppressive phenotype and functional activity in these cells. Leukemic EVs containing 4-1BBL can enhance the suppressive activity and phenotype of Tregs through the regulation of mTOR and STAT5 signaling pathways, resulting in the inhibition of CD8^+^ T cell proliferation (124). In summary, these studies provide evidence that TDEs can modulate the immune system by influencing Tregs, ultimately leading to increased immunosuppression of CD8^+^ T cells. Moreover, targeting of Tregs by inhibiting TDEs is a promising approach for tumor treatment.

#### 2.2.5 Other cells

In addition to the aforementioned cell populations, other cell types exert a significant influence on regulating CD8^+^ T cells. In recent years, EVs have emerged as key players in these processes.

B cells play a significant role in the TME, and recent studies have revealed intriguing findings regarding the impact of TDEs on CD8^+^ T cell function through their influence on these cells (125). Specifically, these studies have emphasized the role of exosomal placenta growth factor (PlGF), a member of the vascular endothelial growth factor family, released by glioma cells. PlGF has been observed to induce the generation of regulatory B cells (Bregs), subsequently suppressing the activities of CD8^+^ T cells. Naïve B cells uptake PlGF, promoting their differentiation into TGF-β^+^ Bregs. This process results in the suppression of CD8^+^ T cell proliferation and a reduction in the release of granzyme B and perforin (126, 127). Additionally, Li et al. revealed that microvesicles derived from esophageal cancer carry matrix metalloprotease-9 (MMP9), which converts latent transforming growth factor-β (LTGF-β) to its active form. Consequently, B cells transform into TGF-β^+^ Bregs, thereby suppressing CD8^+^ T cell activities. These mechanisms potentially contribute to tumor evasion of immune surveillance and facilitate tumor growth (128). Overall, TDEs have the ability to transfer cargo to B cells, inducing their conversion into Bregs, significantly impacting the inhibition of CD8^+^ T cell function.

CD4^+^ T cells, a subset of T lymphocytes characterized by the expression of the CD4 molecule on their cell surface, play a crucial role in antitumor immune responses by either enhancing or suppressing cytotoxic T cell activities (129). Although research in this area is limited, studies have reported that CD4^+^ T cells uptake TDEs, leading to disruptions in the functioning of CD8^+^ T cells. In malignancies involving fluid accumulation, HSP90α presented on the surface of TRAPs stimulates CD4^+^ T cells to produce IL-6 and IL-10 through a signaling cascade involving TLR2-MyD88-NF-κB. Importantly, CD4^+^ T cells activated by these TRAPs suppress the IFN-γ response of both CD4^+^ and CD8^+^ effector T cells through the action of IL-6 and IL-10. Consequently, these processes promote tumor growth and metastasis (130).

In summary, TDEs play a significant role in various mechanisms utilized by tumors to evade detection and immune attacks. Tumor antigens carried by TDEs hinder the cross-presentation of DCs and the expression of maturation markers, impair CD8^+^ T cell activation, and promote the development of tumors ultimately. TDEs that carry specific miRNA could trigger the polarization of macrophages into an M2-like phenotype and upregulate the expression of PD-L1, which reduce activity of CD8^+^ T cells and lead to enhancing tumor growth. TDEs with specific cargo could regulate the differentiation and phenotype of MDSCs and lead to the amplification and activation of MDSCs, which would inhibit CD8^+^ T cells and promote tumor growth. TDE carries specific biomolecules to promote Tregs, thereby inhibiting the immune function of T cells and achieving the purpose of promoting tumor development. These mechanisms suggest that TDE affects the growth of cells in the TME by carrying specific biological information molecules, thereby inhibiting the immune response of CD8^+^ T cells and thus promoting tumor growth. However, research on the impact of TDEs on cells within the TME lags behind studies focused on CD8^+^ T cells. It is crucial to investigate the functional molecules carried by TDEs to gain a comprehensive understanding of how tumor cells influence CD8^+^ T cells through interactions with TME cells. Furthermore, the functional molecules carried by TDEs have the potential to be targets for tumor immunotherapy, providing guidance for tumor treatment.

## 3 Effects of CD8^+^ T cell-derived EVs on tumors

**Figure 3:**
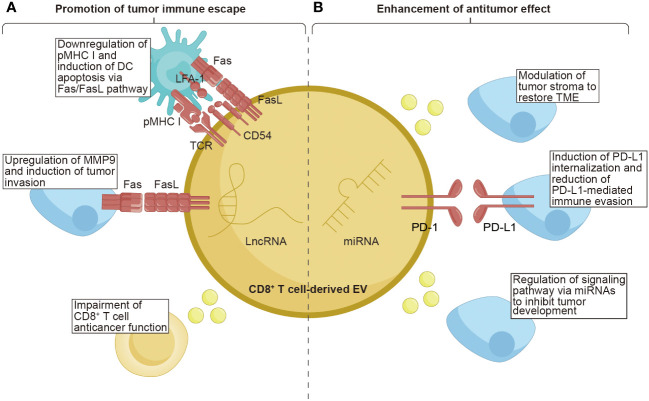
The function of CD8^+^ T cell-derived EVs on tumors. CD8^+^ T cell-derived EVs possess the unique properties of both promoting tumor immune escape and enhancing the anti-tumor effect within tumor tissues. **(A)** CD8^+^ T cell-derived EVs carrying TCR and FasL lead to the downregulation of pMHC I and induce DCs apoptosis via the Fas/FasL pathway. The FasL, as the ligand for Fas, can upregulate MMP9, promoting tumor invasion. Moreover, EVs from exhausted CD8^+^ T cells containing lncRNAs have been demonstrated to impair the anticancer function of CD8^+^ T cells. **(B)** CD8^+^ T cell-derived EVs contain miRNAs that exert a role in modulating the tumor stroma, restoring the TME, and regulating signaling pathways to inhibit tumor development. Additionally, the PD-1 molecules present on the surface of EVs derived from CD8^+^ T cells can induce internalization of PD-L1, thereby reducing PD-L1-mediated immune evasion. TCR, T cell receptor; pMHC I, peptide major histocompatibility complex class I; FasL, Fas ligand; LFA-1, lymphocyte function-associated antigen-1; PD-1, programmed cell death protein 1; PD-L1, programmed cell death ligand-1; MMP9, matrix metalloprotease-9; LncRNAs, long non-coding RNAs; TME, tumor microenvironment.

In the cancer-immunity cycle, CD8^+^ T cells are widely perceived to directly engage and eliminate tumor cells through contact. Moreover, during the initiation of tumors, CD8^+^ T cells have the capability to release EVs in response to this event. CD8^+^ T cell-derived EVs exhibit a dual nature, simultaneously promoting tumor evasion while enhancing antitumor immune responses (**Figure 3** and **Table 2**).

**Table 2:** Summary of EV cargo in CD8^+^ T cell-derived EVs on tumors.

EV cargo	Target cell	Mechanism	Function	Ref.
TCR, FasL, CD54	DC	Downregulate pMHC I and FasL-mediated cytotoxicity	Inhibit CD8^+^ T cell responses	(131)
FasL	Melanoma and lung tumor cell	Activate Fas signaling pathway to upregulate MMP9 expression by inducing c-FLIP accumulation and activating ERK and NF-κB pathways	Promote tumor cell invasion	(132)
LncRNAs	Normal CD8^+^ T cell	–	Regulate normal CD8^+^ T cell behavior	(133)
MiR-298-5p	Mesenchymal tumor stromal cell	–	Trigger mesenchymal tumor stromal cell apoptotic depletion	(134)
PD-1	Triple-negative breast cancer	Induce PD-L1 internalization through clathrin-mediated endocytosis	Attenuate PD-L1 induced tumor-specific cytotoxic T cell suppression	(135)
MiR-765	Estrogen-driven endometrial cancer	Regulate miR-765/PLP2 axis	Limit estrogen-driven endometrial cancer	(136)

TCR, T cell receptor; FasL, Fas ligand; DC, dendritic cell; pMHC I, peptide major histocompatibility complex class I; MMP9, matrix metalloprotease-9; LncRNAs, long non-coding RNAs; PD-1, programmed cell death protein 1; PD-L1, programmed cell death ligand-1.

### 3.1 Contribution to immune evasion of tumors

Although still limited, some intriguing reports have indicated that CD8^+^ T cells can secrete EVs as one of the mechanisms through which tumors evade the immune system. Targeting the disruption of the Fas/FasL axis represents a focal point for EVs derived from CD8^+^ T cells. Xie et al. found that DCs uptook exosomes secreted by CD8^+^ T cells, expressing TCR and FasL on their surface. This uptake led to the interaction between pMHC I-TCR and CD54-LFA-1 on both DCs and the absorbed exosomes. Consequently, the expression of pMHC I in DCs was downregulated, and the DCs were susceptible to cell death through the Fas/FasL pathway. As a result, the antitumor CD8^+^ T cell response stimulated by DCs was inhibited (131). Another study showed that activated CD8^+^ T cells could generate exosomes that carried FasL. These exosomes could promote the invasion of melanoma and lung tumor cells by activating the Fas signaling pathway to upregulate MMP9 expression. Specifically, exosomes derived from activated T cells induced the accumulation of c-FLIP, an endogenous inhibitor of apoptotic death, causing the activation of the ERK and NF-κB pathways. This activation resulted in an increased expression of MMP9 (132).

Continuous antigenic stimulation of CD8^+^ T cells leads to their exhaustion, resulting in a decline in their functional capacity and proliferation ability (137). Additionally, exhausted CD8^+^ T cells can release EVs that have detrimental effects on normal T cells. Wang et al. revealed that normal CD8^+^ T cells are capable of internalizing exosomes released by exhausted CD8^+^ T cells, compromising their anticancer function. Furthermore, the exosomes derived from exhausted CD8^+^ T cells were found to harbor long noncoding RNAs implicated in modulating the behavior of normal CD8^+^ T cells. These findings contribute to our understanding of the varied states of CD8^+^ T cells and provide valuable insights into dysfunctional anticancer immunity (133). Overall, these studies confirm the immunosuppressive role of CD8^+^ T cell-derived EVs in tumor progression.

### 3.2 Enhancement of antitumor effects

Notably, CD8^+^ T cell-derived EVs exhibit dual effects, as they can both promote tumor metastasis and cancer evasion, as well as enhance antitumor immunity. The tumor stroma, a vital part of the TME, is composed of cells and noncellular connective tissue that provide structural support, exerting a crucial effect on the initiation, progression, and metastasis of tumors (138). Therefore, modulating the tumor stroma is considered an effective approach to restore the TME. Seo et al. revealed that activated CD8^+^ T cells isolated from healthy mice released cytotoxic EVs containing miR-298-5p, capable of triggering apoptotic depletion of mesenchymal tumor stromal cells. This mechanism effectively inhibited tumor invasion and metastasis (134). Interfering with the interaction between PD-1 and PD-L1 is a crucial factor in enhancing the cytotoxic potential of CD8^+^ T cell-derived EVs. Qiu et al. discovered that exosomal PD-1 derived from activated T cells played a role in attenuating PD-L1-induced immune evasion in triple-negative BC. This effect was achieved by inducing PD-L1 internalization through clathrin-mediated endocytosis suggesting that PD-1 molecules had a favorable influence on antitumor immune response (135). Manipulating signaling pathways through miRNA modulation serves as a powerful strategy employed by CD8^+^ T cells to inhibit tumor development. Zhou et al. found that the downregulation of miR-765 promoted cell proliferation, epithelial-mesenchymal transition, and invasion in uterine corpus endometrial cancer by activating the PLP2-Notch signaling pathway. Estrogen/ERβ, a known risk factor for uterine corpus endometrial cancer, regulated the miR-765/PLP2 axis to accelerate disease progression. However, it was observed that CD45RO^-^CD8^+^ T cell-derived EVs effectively ameliorated estrogen-driven endometrial cancer development by releasing significant amounts of miR-765 (136). The aforementioned studies indicate that EVs derived from CD8^+^ T cells can enhance their antitumor capabilities through various mechanisms. These findings highlight the unexplored avenues for CD8^+^ T cell-derived EVs in cancer therapy, further enriching their potential applications in the field.

It is worth mentioning that CTLs have the ability to secrete exosomes. Upon stimulation by IL-12, these exosomes can activate bystander CD8^+^ T cells, similar to observations made during infections (139). Additionally, CTL-derived exosomes can facilitate the activation of CTLs through low-affinity peptides in the presence of IL-12 (140). However, further investigation is warranted to explore these processes within the context of tumors.

## 4 Application of EVs to activate CD8^+^ T cell antitumor response

Exosomes are stable and have slow clearance with low toxicity and immunogenicity in circulation, moreover, they can spread in tumor tissues and pass the physical barriers like the blood-brain barrier (141). These characteristics of exosomes give them a natural advantage in tumor therapy. We classify exosome-mediated tumor therapy into two categories: tumor-targeted therapy and tumor immunotherapy.

### 4.1 Tumor-targeted therapy

In targeted tumor therapy, exosomes serve as carriers to deliver targeted therapeutic drugs to tumors. They can stably carry oncologic drugs to avoid enzymatic degradation of drugs, prolong the half-life of drugs during delivery, and target specificity with extremely high bioavailability (141, 142). It has been established that homotypic adhesion molecules on exosome membranes endow exosomes with strong preferential binding to the source cells (143). In addition, MSC-Exo was more preferred to be uptake by cancer cells in tumor tissue (144–146). The homing properties of exosomes make them effective vectors for tumor targeting. As an excellent carrier, exosomes can carry a variety of tumor treatment drugs (like chemotherapeutic drugs (147), radiosensitizing agents (148), photothermal agents (149), sonosensitizers (150), siRNA (151), miRNA (152), and CRISPR/Cas 9 (153)), providing more possibilities for tumor treatment (chemotherapy, radiotherapy, photodynamic therapy, photothermal therapy, sonodynamic therapy, and gene therapy). At present, in order to achieve more efficient tumor treatment, many multifunctional exosome therapy schemes have been designed. Wang et al. developed a multifunctional exosome tumor therapy platform (Exo-DOX-Fe_3_O_4_@PDA-MB) that combined chemotherapy, gene therapy, and photothermal therapy. Doxorubicin (DOX) was encapsulated into the isolated exosomes by electroporation, and the exosome membrane was coated with Fe_3_O_4_ which was modified with molecular beacon (MB) through polydopamine. The Exo-DOX- Fe_3_O_4_@PDA-MB were enriched at the tumor site by external magnetic field guidance. Then the application of near-infrared radiation (NIR) induced localized hyperthermia and triggered the release of DOX loaded inside the exosome, which led to about 91.04% of cancer cells being killed. The released MB could target the miR-21 for both imaging and gene silencing. However, there are still some disadvantages with the native exosomes. To improve the effect of tumor treatment and reduce adverse effects, more and more exosome modification methods have been developed to construct functional exosomes recently (154). Tao et al. engineered exosomes with HER2 ligand (CAR) by biological modification and then inserted transferrin receptor binding peptide modified with DSPE-PEG (DSPE-PEG-T7) into the exosome membrane through hydrophobic action. The obtained ExoCAR/T7 could not only accurately target HER2+ breast cancer cells but also increase the penetration rate of the blood-brain barrier (155). One more, Cho et al. utilized membrane-bound cytokine technology to genetically modify human primary CD8^+^ T cells, resulting in engineered EVs containing IL-2 and an anti-EGFR antibody on the surface. Each tether molecule of these engineered EVs enhanced tumor cytotoxicity and promoted cancer targeting (156).

### 4.2 Tumor immunotherapy

Tumor immunotherapy is a novel anti-cancer strategy that activates immune cells, unlike tumor-targeted therapy which primarily targets tumors themselves, these activated immune cells can spread tumor information to other unactivated immune cells throughout the body, and even if tumor cells try to spread to other sites, these primed immune cells can detect and clear them, providing a whole-body defense system for cancer (157, 158). CD8^+^ T cells play a crucial role in cancer immunotherapy as they are specifically targeted to destroy tumor cells. Among them, the most effective adoptive CD8^+^ T cell treatment for B cell lymphomas is anti-CD19 chimeric antigen receptor T cell therapy (159). EVs have emerged as a novel cancer immunotherapy in recent years owing to the rapid advancement of cancer research. The exosomes that mediate tumor immunotherapy include tumor cell-derived exosomes, immune cells-derived exosomes, and engineered exosomes. Here we focus on DC- and IC-mediated EV treatment which activate CD8^+^ T cells for tumor immunotherapy (**Table 3**).

**Table 3:** CD8^+^ T cell-based EV applications.

EV		Mechanism of action	Effects on CD8^+^ T cells	Type	Ref.
Source	Cargo				
DC-mediated					
Dex	MUC1 glycopeptide	Promote DC maturation and antigen presentation by inducing CD80/CD86 and MHC I/MHC II expression respectively	Enhance CD8^+^ T cell cytotoxicity	Melanoma	(160)
Dex	RAE-1γ	Dependence of NKG2D/NKG2DL (RAE-1γ)	Promote CD8^+^ T cell activation and proliferation	Chronic myeloid leukemia	(161)
Dex	A-PaschiRNA	Promotion of antigen cross-presentation capacity	Induce CD8^+^ T cell proliferation and IFN-γ secretion	Esophageal cancer	(162)
TMV	MUC1 tumor glycoantigen	Restore the pH of the phagosome to near-neutrality; Promote cross-processing of the MUC1 antigen	Stimulate IFN-γ response of CD8^+^ T cells	Ovarian cancer	(163)
TDE	CD80 and CD86	Promote the phenotypic and functional maturation of DCs by enhancing the expression of CD80 and CD86	Induce antigen-specific CTL response	Leukemia	(164)
TDE	Let7i and miR-142	Promote DC maturation	Promote IFN-γ and granzyme B production in CTLs	Breast cancer	(165)
TDE	MiR-155	Promote DC maturation and activation	Accelerate the differentiation, proliferation, cytotoxicity effects, and infiltration into the TME of CTLs	Colorectal cancer	(166)
TDE	TGF-β1	Reduce TGF-β1 expression; Promote DC maturation and function	Induce antigen-specific CTL response	Leukemia	(167)
TDE	PD-L1	Downregulate PD-L1 expression; Induce DCs maturation	Promote T cell activation and proliferation; Induce antigen-specific CTL response	Leukemia	(168)
Functionalize Dex	anti-CTLA-4 antibody	Block of CTLA-4 checkpoint	Induce T cell activation and proliferation; Improve tumor homing of effector T cells	Melanoma	(169)
α-LA engineered TDE	Hiltonol and ELANE	Induce immunogenic cell death; Promote cDC1 antigen cross-presentation	Induce tumor-reactive CD8^+^ T cell responses	Breast cancer	(170)
Engineered sEV	PH20 hyaluronidase	Penetrate tumor foci by HA degradation; Activate DCs maturation and migration	Activate tumor-specific CD8^+^ T cells	Melanoma and breast cancer	(171)
Immune checkpoint-based					
TDE	PD-L1	Inhibit TDE PD-L1 secretion by MAC targeting ETA	Enhance CD8^+^ T cell-mediated tumor killing	Breast cancer	(172)
sEV	PD-L1	Inhibit sEV and cellular PD-L1 levels by temsirolimus	Promote CD8^+^ T cells proliferation and activation	Breast cancer	(173)
TDE	PD-L1	Inhibit TDE secretion and exosomal PD-L1 via SFX	Increase the number of CTLs	Breast cancer and colorectal cancer	(174)

Dex, DC-derived EVs; RAE-1γ, retinoic acid early inducible-1γ; NKG2DL, NKG2D ligand; A-PaschiRNA, ASTN2-PAPPA_antisense_ chiRNA; TMV, Tumor-derived microvesicles; TDE, tumor-derived extracellular vesicle; TME, tumor microenvironment; α-LA, α-lactalbumin; sEV, small EVs; DC, dendritic cell.

#### 4.2.1 DC-mediated EV treatment

DCs play a crucial role as mediators of immune responses by capturing, processing, and presenting antigens to CD8^+^ T cells. This interaction constitutes an essential function in the activation, proliferation, and differentiation of CD8^+^ T cells, ultimately leading to the elimination of tumor cells and the establishment of long-term immunity. Given the unique characteristics of DCs, DC-mediated EV therapy is emerging as a novel approach in cancer treatment. On one hand, EVs released by DCs directly activate CD8^+^ T cells, stimulating them to secrete cytokines and cytolytic molecules that contribute to tumor eradication. On the other hand, TDEs act indirectly through DCs to facilitate CD8^+^ T cells in killing tumor cells.

**Figure 4:**
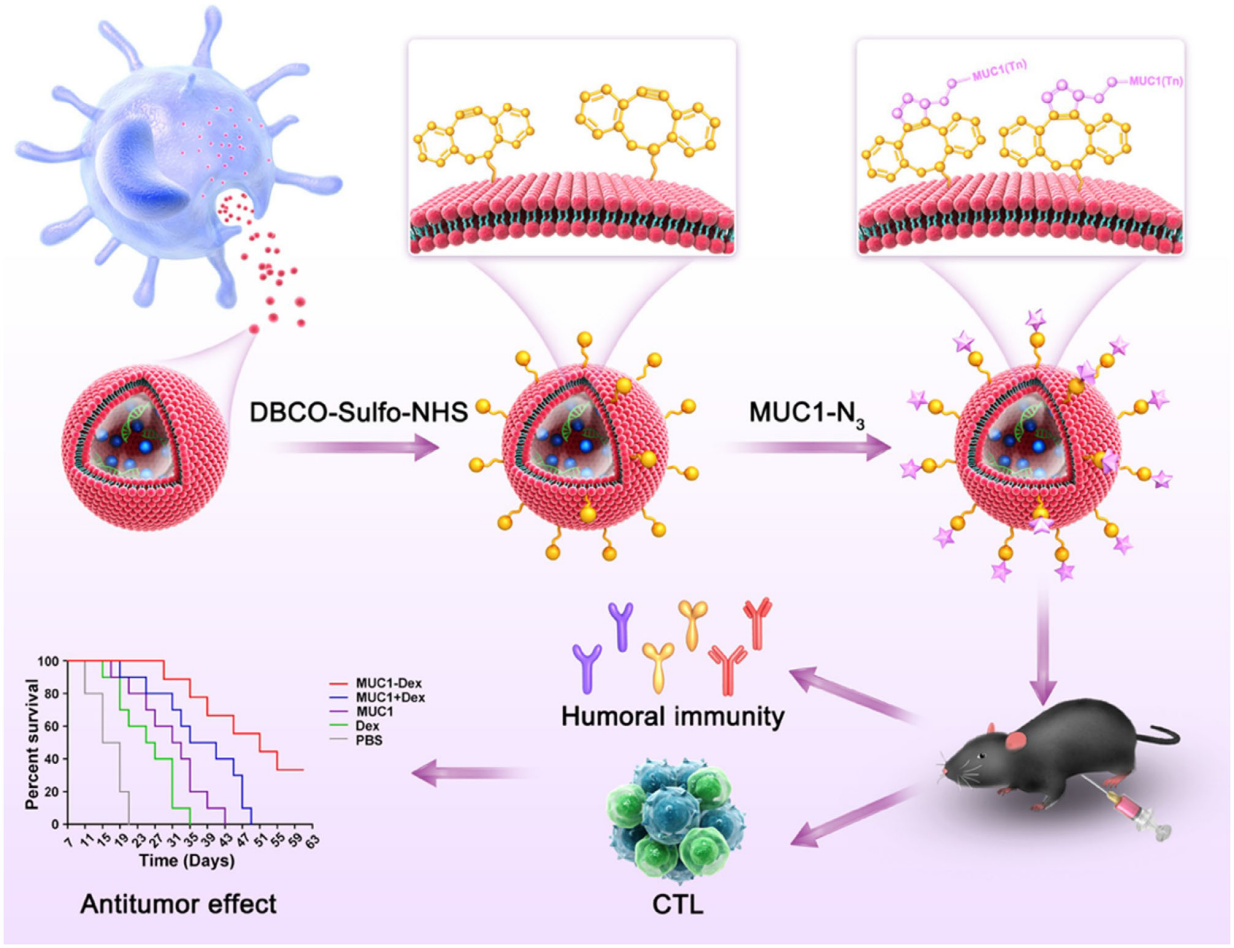
Schematic diagram of the preparation of MUC1 glycopeptide-Dex conjugate vaccines and the enhancement of CTL cytotoxicity (160). Copyright(2022), with permission from Acta Biomaterialia. CTL, cytotoxic CD8^+^ T cell.

**Figure 5:**
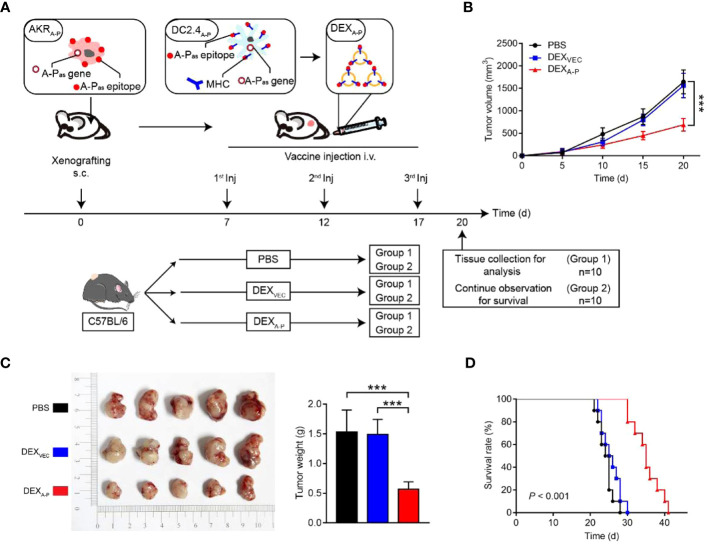
Therapeutic effect of DEX_A-P_ vaccination in esophageal cancer model. **(A)** DEX_A-P_ were i.v. injected in AKR_A-P_ tumour-bearing mice. Treatment scheme indicates the timing of tumour inoculation and repeated vaccination of C57BL/6 mice. **(B)** Tumour growth curve after vaccination with DEX_A-P_, DEX_VEC_ or PBS. **(C)** Representative images (left panel) and average weights (right panel) of tumours harvested on 20 d after AKR_A-P_ cancer cell inoculation. **(D)** Kaplan-Meier survival curves of tumour-bearing mice vaccinated with DEX_A-P_, DEX_VEC_ or PBS (162). Copyright(2022), with permission from Journal Of Extracellular Vesicles. *** p<0.001; DEX_A-P_, ASTN2-PAPPA_antisense_ chiRNA-loaded dendritic cell-derived extracellular vesicle.

Currently, DC-derived exosomes (Dex) are being employed as an effective and promising antitumor strategy for CD8^+^ T cell-mediated immunotherapy. Dex vaccines, considered a novel form of immunotherapy, carry various molecules to exert their immune effects (175). Notably, proteins play a crucial role in Dex vaccines, significantly contributing to their ability to modulate the immune response. An example is the utilization of MUC1 glycopeptide-Dex conjugate vaccines, which enhance the antigen presentation capacity of DCs (**Figure 4**). These vaccines stimulate the generation of antigen-specific CTLs capable of effectively eliminating tumors and delaying tumor development (160). Ligands, which are proteins that bind to other biomolecules and have biological activities, serve as important regulators of Dex functions. Dex vaccines containing retinoic acid early inducible-1γ, a ligand of NKG2D, have demonstrated the capacity to effectively enhance the proliferation and effector functions of CD8^+^ T cells. In a mouse model of chronic myeloid leukemia, this vaccine not only exhibited potent therapeutic effects but also induced durable immune memory (161). Chimeric RNA (chiRNA), composed of exons from two or more different genes, can encode novel proteins, thereby altering cellular phenotypes (176). In recent years, chiRNA has been explored for use in Dex vaccines. For instance, the utilization of ASTN2-PAPPA_antisense_ chiRNA-loaded Dex (DEX_A-P_) in cancer vaccination has shown promising results. This approach promotes antigen cross-presentation, thereby activating CD8^+^ T cells. *In vitro* experiments have indicated that DEX_A-P_ vaccination prolongs the survival of mice with esophageal cancer (**Figure 5**), suggesting that transcription-induced chiRNAs could serve as a source of cancer-specific and mutation-independent neoantigens, demonstrating substantial potential for the treatment of malignancies with low mutational burden or lacking mutation-based antigens (162). Improving antigen presentation poses indeed a significant challenge in the development of Dex-based cancer vaccines. Identifying suitable cargo that effectively enhances antigen presentation and promotes robust immune responses, remains crucial. Determining the appropriate cargo for Dex vaccines is a vital direction for future research, contributing to the advancement of the field and enhancing the effectiveness of cancer immunotherapy approaches.

In addition to the previously mentioned strategies, an alternative approach with promising potential involves utilizing TDEs to stimulate CD8^+^ T cell-mediated immune responses by acting on DCs. Similarly, an effective method for enhancing antitumor immunity mediated by DCs through TDEs is to regulate the cargo carried by these vesicles. A potential strategy involves loading tumor-associated antigens onto TDEs. Microvesicles derived from tumors, carrying the MUC1 tumor glycoantigen, could be utilized to pulse DCs and activate an IFN-γ response mediated by MUC1-specific CD8^+^ T cells, ultimately promoting an antitumor response (163). Promising results have been observed in enhancing the antigen presentation of DCs by upregulating costimulatory molecules in TDEs. In the case of leukemia, exosomes derived from leukemia cells could be generated using lentiviral vectors encoding CD80 and CD86, two B7 costimulatory molecules. These exosomes exhibit high CD80 and CD86 expression levels. When administered to DCs, they increase the expression of CD80 and CD86, leading to the induction of CTL responses. Consequently, this approach enhances antileukemia immunity (164). Modifying TDEs using miRNAs holds potential for various applications. TDEs loaded with let7i and miR-142 have been shown to promote DC maturation and induce the release of a significant amount of granzyme-B from CD8^+^ T cells, proving to be effective in suppressing BC (165). The enrichment of miR-155 in TDEs has been observed to promote the differentiation, proliferation, and cytotoxicity of CTLs through DCs. This enhanced immune response effectively controls tumor growth and improves overall survival (166). Furthermore, TDEs can induce DCs to enhance the antitumor effectiveness of CTLs by silencing specific molecules. For instance, in the case of leukemia, exosomes derived from tumor cells with silenced TGF-β1 can effectively promote DC maturation by reducing TGF-β1 expression, resulting in a more effective induction of tumor-specific CTL responses (167). Additionally, exosomes derived from tumor cells with silenced PD-L1 could induce improved DC maturation, consequently leading to antigen-specific CTL responses (168). These studies highlight the feasibility of enhancing the antitumor immunity of DCs through the regulation of molecules on EVs. This approach offers a potential therapeutic strategy to facilitate tumor immunotherapy, aiming to improve treatment outcomes.

**Figure 6:**
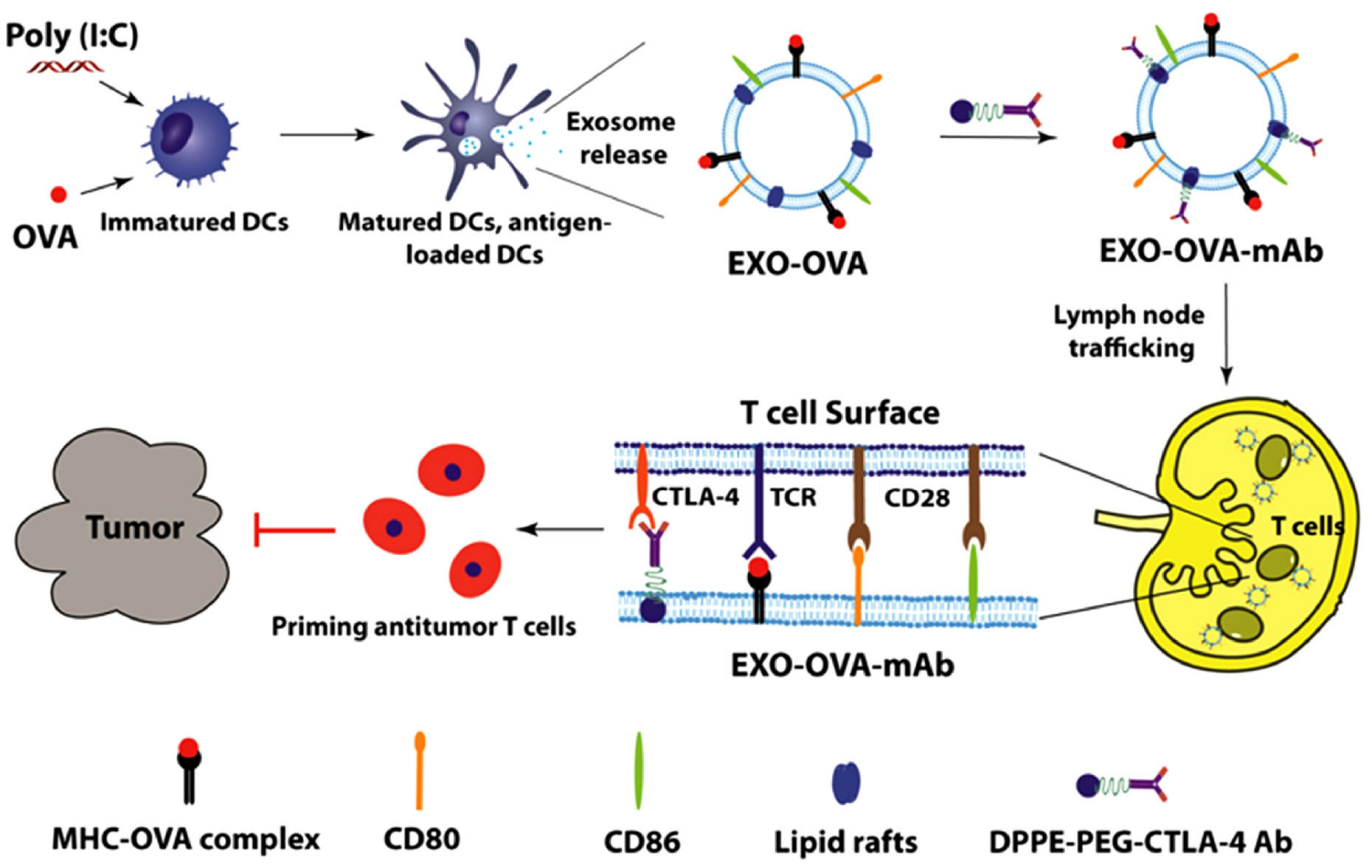
Schematic diagram of the preparation of anti-CTLA-4 antibody-functionalized Dex and the induction of tumor-specific T-cell responses (169). Copyright(2020), with permission from Acta Biomaterialia. Dex, DC-derived EVs.

**Figure 7:**
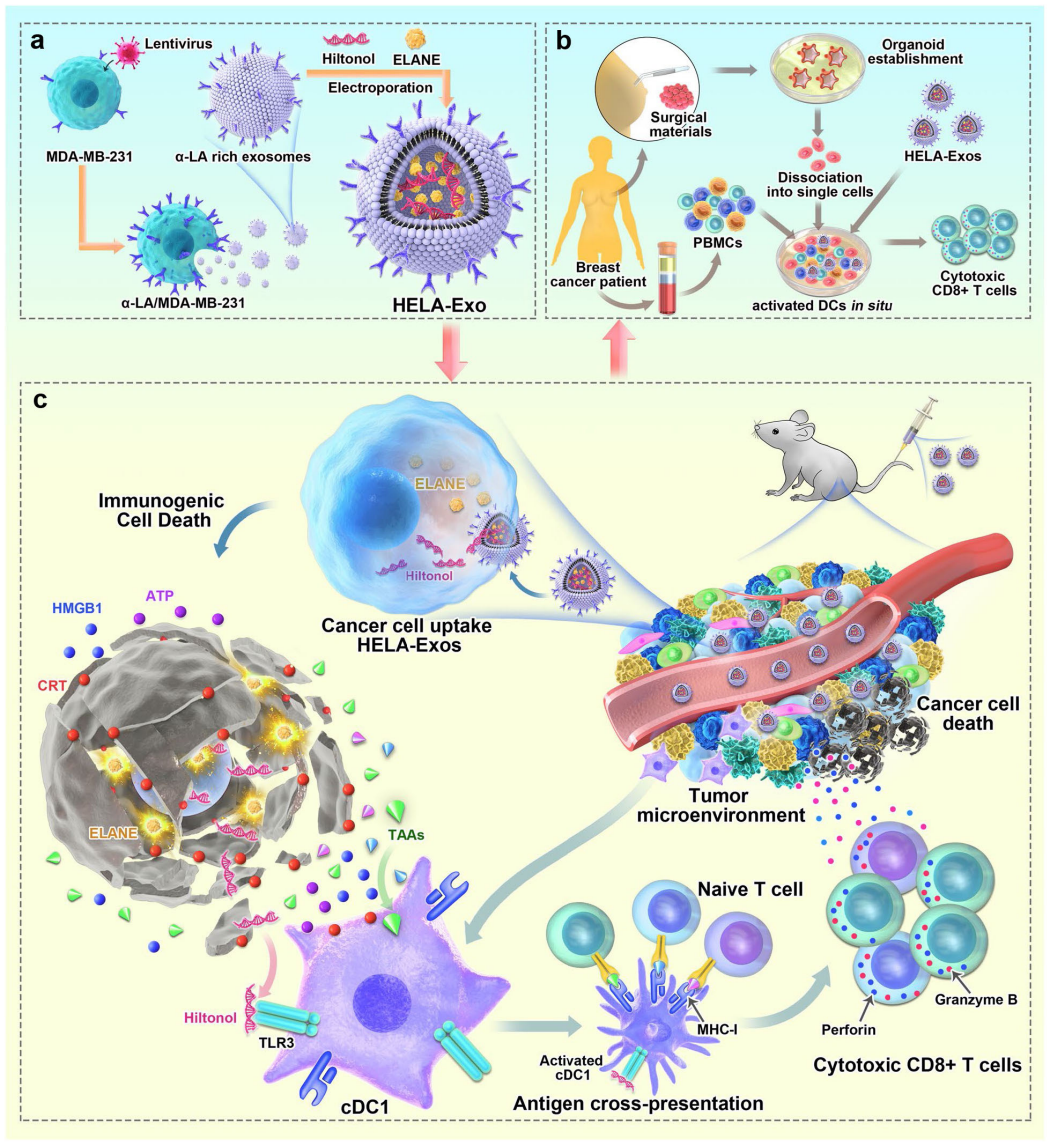
Schematic diagram of the preparation of HELA-Exos and their application as an *in situ* DC-primed vaccine for BC treatment. **(A)** The preparation of HELA-Exos. **(B)** The mechanism of HELA-Exos which activate DCs *in situ* in a TNBC mouse xenograft model. **(C)** The activation of DCs *in situ* by HELA-Exos in the PBMC-autologous tumor organoid coculture system of BC patients (170). Copyright(2022), with permission from Molecular Cancer. HELA-Exos, Hiltonol-ELANE-α-LA-engineered exosomes; BC, breast cancer; DC, dendritic cell; TNBC, triple-negative breast cancer; PBMC, peripheral blood mononuclear cells.

Furthermore, alternative approaches for designing EVs to fulfill a similar function are being explored. One such method involves combining engineering techniques with exosomes. Studies have demonstrated that using a lipid-anchoring technique to functionalize Dex membranes with an anti-CTLA-4 antibody could effectively enhance the CTL/Treg ratio (**Figure 6**), ultimately initiating antitumor T cell responses and inhibiting tumor growth (169). Loading specific cargo into engineered EVs is indeed a viable option. For example, Huang et al. loaded Hiltonol (a TLR3 agonist) and ELANE (human neutrophil elastase, an inducer of immunogenic cell death) into exosomes engineered with α-lactalbumin. The engineered exosomes displayed tremendous potential to enhance the activation of type one conventional DCs in their original location, subsequently leading to the cross-priming of tumor-reactive CD8^+^ T cell responses (**Figure 7**) and resulting in potent inhibition of tumor growth (170). It is well established that low molecular weight oligo hyaluronan can activate DCs. In this context, engineered EVs carrying PH20 hyaluronidase could facilitate DC maturation and migration. Importantly, these EVs have a particular affinity for CD103^+^ DCs, which playing an essential part in activating immune responses of tumor-specific CD8^+^ T cells. Consequently, this immune activation leads to the suppression of tumor growth (171). Collectively, these approaches offer valuable and innovative insights into the antitumor effects achieved through the utilization of DC-mediated engineered EVs. They effectively demonstrating the therapeutic potential of engineered EVs for treating tumors.

#### 4.2.2 IC-based EV treatment

IC therapy (ICT) has revolutionized anticancer immunotherapy by disrupting the PD-1/PD-L1 interaction and rejuvenating exhausted CD8^+^ T cells, thereby increasing the antitumor immune response (7). ICT has significantly influenced the landscape of cancer treatment, with several IC-based drugs being approved for various cancers (177). However, it is essential to note that only a subset of patients with specific cancers respond positively to ICT (178, 179). The limited efficacy of ICT in certain cases has prompted researchers to investigate the underlying mechanisms. Interestingly, the immunosuppressive role of EVs has motivated researchers to explore their potential association with ICs.

Currently, there is significant research interest in exploring the correlation between EVs and PD-1/PD-L1. It has been reported that increased levels of PD-L1 on TDEs could inhibit the function of CD8^+^ T cells and promote cancer growth. This discovery served as the foundation for investigating the potential therapeutic targeting of exosomal PD-L1 (71). In the context of melanoma tumors, HRS phosphorylation restricted the migration of CD8^+^ T cells toward the tumor location by releasing inhibitory EVs containing PD-L1. This regulatory mechanism played a role in modulating anticancer immunity. Consequently, blocking HRS phosphorylation showed promise as a potentially novel solution for tumor immunotherapy (180).

**Figure 8:**
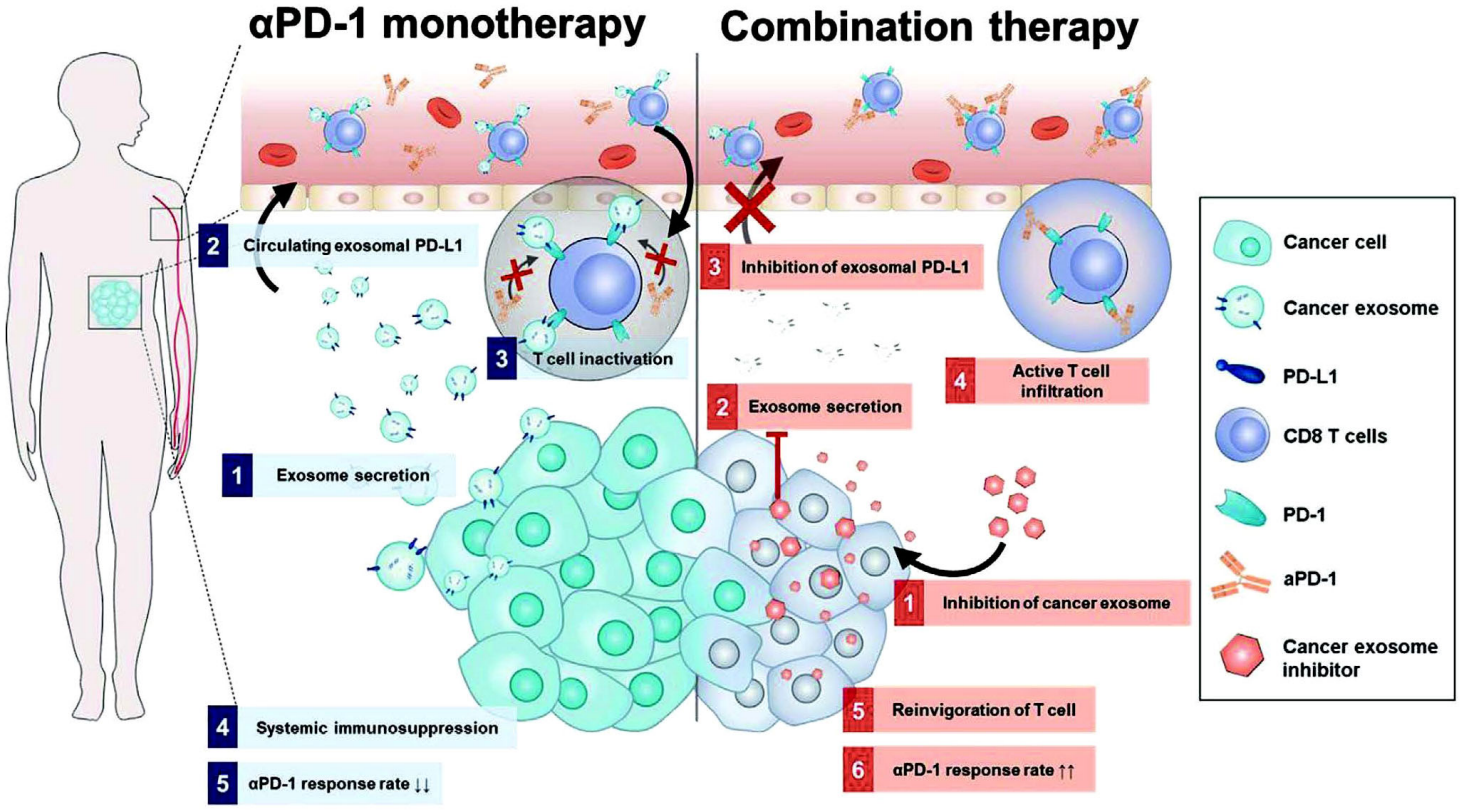
Mechanism diagram of combination treatment using sulfisoxazole and aPD-1. Sulfisoxazole suppresses tumor exosome biogenesis and cooperatively improves aPD-1 antitumor effect. 1) Tumors secrete exosomal PD-L1, which suppresses T cell activation to evade aPD-1 monotherapy immunity. 2) Sulfisoxazole suppresses the biogenesis of exosomes in tumors, thus improving aPD-1 antitumor efficacy (174). Copyright(2022), with permission from Advanced Science. Abbreviations: aPD-1, anti-PD-1.

​To overcome limitations observed in ICT for tumors, researchers have made significant progress in identifying drugs that interfere with the PD-1/PD-L1 interaction. One such drug is macitentan, which is administered orally. Macitentan has been found to limit the secretion of PD-L1 by TDEs through its action on endothelin receptor A in BC cells. By attenuating the PD-1 interaction with TDE PD-L1, macitentan enhances the tumor-killing effects of CD8^+^ T cells. This discovery suggests that macitentan could potentially improve the limited response observed in PD-1/PD-L1 blockade therapy (172). Recent research findings indicate that temsirolimus could evidently limit the secretion of PD-L1 by EVs through the activation of autophagy, helping overcome the limited responsiveness observed in ICT (173). Furthermore, sulfisoxazole has also been identified to be capable of inhibiting the biogenesis of exosomes with PD-L1, thus improving the antitumor effect of anti-PD-1 monotherapy (**Figure 8**) (174). These findings provide potential avenues for enhancing the therapeutic efficacy of ICT by targeting EV-mediated PD-L1 signaling. However, the precise mechanisms by which these drugs affect EV PD-1/PD-L1 interactions and the role of the TME in this process require further investigation. It is also crucial to identify drugs targeting EV PD-1/PD-L1 that can be translated into clinical use. The exploration of such drugs has become a priority for researchers in this field. Additionally, it is important to investigate the immunosuppressive effects of IC molecules on EVs derived from other cell types and develop drugs to counteract these effects.

## 5 Conclusions

The role of EVs in TME, as an important communication mediators between cells, has become a focus of research in recent years. Previous studies have primarily focused on elucidating the bidirectional communication between CD8^+^ T cells and tumor cells, as well as between various cells of the TME and tumor cells (47, 181, 182). However, we believe that EVs, serving as pivotal information carriers, exert influence not only on limited bidirectional communication but also on the intricate immunomodulatory network involving CD8^+^ T cells, TME, and tumor cells. Thus, this review provides insights into the functions performed by EVs in the dynamic interactions among tumors, CD8^+^ T cells, and the TME. It also highlights the utilization of functionalized EVs based on DCs and ICT in cancer immunotherapy. In particular, TDEs can directly or indirectly suppress the antitumor immune response mediated by CD8^+^ T cells. This occurs through the delivery of bioactive molecules, including proteins, miRNA, and circRNA, either on their surface or within their cargo. Consequently, CD8^+^ T cell activation, proliferation, differentiation, and function are inhibited, whereas promoting apoptosis and exhaustion are promoted. Furthermore, TDEs also indirectly hinder CD8^+^ T cell function by promoting the proliferation and activation of immunosuppressive cells such as MDSCs and Tregs, polarization of M2 TAMs and Bregs, and inhibition of antigen presentation by DCs. These mechanisms collectively facilitate tumor growth within the TME. Conversely, EVs derived from CD8^+^ T cells exhibit dual regulatory effects on tumors. On one hand, they enhance tumor growth by suppressing the antigen presentation functionality of DCs and promoting tumor cell invasion. On the other hand, through miRNAs and PD-1, these EVs modulate the tumor stroma, restore the TME, and promote PD-L1 internalization, ultimately inhibiting tumor growth. Due to the complexity of TME, numerous unsolved challenges still need to be addressed. The identification of EV origin remains challenging due to the presence of numerous subpopulations. Enhancing technological advancements for determining the source of EV represents a crucial future endeavor. Additionally, our understanding of EVs within the TME, particularly in the context immune cells, remains limited, necessitating further exploration of the intricate interactions between EVs and various immune cells in tumor settings. Targeting these EVs holds promising potential in enhancing the effectiveness of tumor immunotherapy. Conducting more detailed studies will help us better understand the intricate immunomodulatory networks associated with tumors.

Moreover, owing to their unique characteristics, such as traversing biological barriers, transporting molecules, low immunogenicity, and high safety, EVs already show promise as agents for tumor therapy. Based on this information, the review summarizes current common strategies involving functionalized EVs based on tumor-targeted therapy and tumor immunotherapy. We aim to provide new insights into EV-based therapeutic approaches for cancer. In recent years, extensive research on the role of EVs in tumor immunity has opened up diverse possibilities for cancer diagnosis and treatment. By delving into the crucial functions of EVs in regulating tumor immune evasion, activating immune responses, and combating tumor growth, we can achieve a deeper level of understanding and leverage the valuable information and signals carried by these minuscule vesicles. Although there are currently only a few relevant studies on CD8^+^ T cell-derived EVs in cancer, these EVs hold great potential as a novel approach for cancer treatment. However, several unresolved issues persist in EV preparation and study. Primarily, several methods are available for isolating and purifying EVs, making it challenging to establish standardized protocols for their isolation, purification, and characterization (183, 184). Furthermore, identifying the molecular cargo encapsulated within EVs could yield valuable biomarkers for tumor diagnosis and prognosis, presenting new avenues for early tumor detection (185, 186). Lastly, although the significance of EV PD-L1 in immune evasion is recognized, research on drugs that block EV PD-L1 to reactivate CD8^+^ T cells remains limited. Hence, the exploration and development of drugs targeting this specific pathway assume paramount importance in tumor immunotherapy. As of March 1, 2024, there are 191 clinical trials on exosome about tumor research searched on the website https://clinicaltrials.gov, and then only a few on tumor immunotherapy of exosomes. There are some controversial reports on whether tumor-derived exosomes and MSC-derived exosomes suppress or promote tumor growth. Besides, until exosome-based therapies enter clinical practice, there is currently a lack of standardized protocols to ensure consistent production of exosomes. At present, there is still a long way to go to realize the clinical application of exosome tumor therapy. Among them, exosome vaccine is the earliest clinical application of exosome in tumor immunotherapy. The researchers induced an immune response by inoculating patients with recurrent glioblastoma with exosomes released after therapeutic tumor cell death (NCT01550523), and the researchers used DC-based exosomes as a vaccine in combination with cyclophosphamide (mCTX) therapy to treat lung cancer (NCT01159288). Continued investigation on the role of EVs in tumor immunity holds the promise of yielding novel breakthroughs and innovations, ultimately expanding the prospects for improved health and increased survival rates among patients.

## Author contributions

ZH: Writing – review & editing, Writing – original draft. XL: Writing – review & editing. QG: Writing – original draft. YZ: Writing – original draft. LS: Writing – original draft. QC: Writing – review & editing. ST: Writing – original draft. QO: Writing – review & editing. JZ: Writing – review & editing.
